# Identifying Caregiver Availability Using Medical Notes With Rule-Based Natural Language Processing: Retrospective Cohort Study

**DOI:** 10.2196/40241

**Published:** 2022-09-22

**Authors:** Elham Mahmoudi, Wenbo Wu, Cyrus Najarian, James Aikens, Julie Bynum, V G Vinod Vydiswaran

**Affiliations:** 1 Department of Family Medicine Medical School University of Michigan Ann Arbor, MI United States; 2 Institute for Healthcare Policy and Innovation University of Michigan Ann Arbor, MI United States; 3 Department of Biostatistics School of Public Health University of Michigan Ann Arbor, MI United States; 4 Medical School University of Michigan Ann Arbor, MI United States; 5 Department of Learning Health Sciences Medical School University of Michigan Ann Arbor, MI United States

**Keywords:** natural language processing, caregiver, medical notes, Alzheimer, dementia, pragmatic, aging, care planning, health care, elderly care, elderly population, algorithm

## Abstract

**Background:**

Identifying caregiver availability, particularly for patients with dementia or those with a disability, is critical to informing the appropriate care planning by the health systems, hospitals, and providers. This information is not readily available, and there is a paucity of pragmatic approaches to automatically identifying caregiver availability and type.

**Objective:**

Our main objective was to use medical notes to assess caregiver availability and type for hospitalized patients with dementia. Our second objective was to identify whether the patient lived at home or resided at an institution.

**Methods:**

In this retrospective cohort study, we used 2016-2019 telephone-encounter medical notes from a single institution to develop a rule-based natural language processing (NLP) algorithm to identify the patient’s caregiver availability and place of residence. Using note-level data, we compared the results of the NLP algorithm with human-conducted chart abstraction for both training (749/976, 77%) and test sets (227/976, 23%) for a total of 223 adults aged 65 years and older diagnosed with dementia. Our outcomes included determining whether the patients (1) reside at home or in an institution, (2) have a formal caregiver, and (3) have an informal caregiver.

**Results:**

Test set results indicated that our NLP algorithm had high level of accuracy and reliability for identifying whether patients had an informal caregiver (*F*_1_=0.94, accuracy=0.95, sensitivity=0.97, and specificity=0.93), but was relatively less able to identify whether the patient lived at an institution (*F*_1_=0.64, accuracy=0.90, sensitivity=0.51, and specificity=0.98). The most common explanations for NLP misclassifications across all categories were (1) incomplete or misspelled facility names; (2) past, uncertain, or undecided status; (3) uncommon abbreviations; and (4) irregular use of templates.

**Conclusions:**

This innovative work was the first to use medical notes to pragmatically determine caregiver availability. Our NLP algorithm identified whether hospitalized patients with dementia have a formal or informal caregiver and, to a lesser extent, whether they lived at home or in an institutional setting. There is merit in using NLP to identify caregivers. This study serves as a proof of concept. Future work can use other approaches and further identify caregivers and the extent of their availability.

## Introduction

Clinical practice creates a large amount of structured and unstructured data [1,2]. Although the electronic medical record (EMR) has allowed health care systems to collect clinical encounter data, the collection process and reporting are still inefficient. This inefficiency is burdensome for health care workers and providers and may negatively impact patient care [1,2]. Furthermore, a large portion of the data in health care is in a free-text format. The data are entered into the system by multiple individuals (medical students, nurses, social workers, etc) and lack a specific template, are not easily searchable by health care workers, and are not readily available for clinical decision-making. Applying natural language processing (NLP) to medical notes has shown promising results in diagnosing certain conditions [3,4], predicting adverse health events [5,6], and identifying social determinants of health [7].

Systematic collection of caregiver information in EMR is a challenging task [8]. Although caregivers play an essential role in the health and well-being of people with complex care needs, such as those with dementia or a disability [9], health care systems are not equipped to readily identify caregiver availability (or lack thereof), type of care provided, time availability, and other helpful information about caregiver support. Despite the emergence of NLP in health care [10-14], there is a paucity of work examining the pragmatic collection of caregiver information [9].

Approximately 6 million older adults in the United States live with dementia. This number is expected to double by 2050 [15]. Because of more cognitive and physical limitations, compared with other older adults, people with dementia often have complex care management needs, and their well-being depends on their caregivers [16-19]. For example, postdischarge care coordination with a patient’s caregiver may reduce readmission or other adverse health events. It is critical for the health systems to quickly identify and act upon caregiver availability information for patients with complex care needs, particularly after hospital discharge.

In this work, we aimed to provide a proof of concept that NLP can reliably identify caregiver availability and type via medical notes. we examined the following three outcomes: (1) whether a patient lives at home vs in an institution, (2) whether a patient has a formal caregiver (paid), and (3) whether a patient has an informal caregiver (eg, a family member). We hypothesized that using NLP, we would be able to reliably determine each of the above outcomes.

## Methods

### Data Source

To examine caregiver availability and type of caregivers, if any, for patients diagnosed with dementia, we used medical notes from Michigan Medicine (MM)a large academic medical center in Southeast Michigan. Our initial patient cohort was identified using the International Classification of Disease, 10th revision codes (Table S1 in Multimedia Appendix 1) from structured EMRs between October 2015 and January 2020. Using a 1-year look back period, we identified 2205 unique patients with dementia with at least one hospitalization in MM.

**Figure 1:**
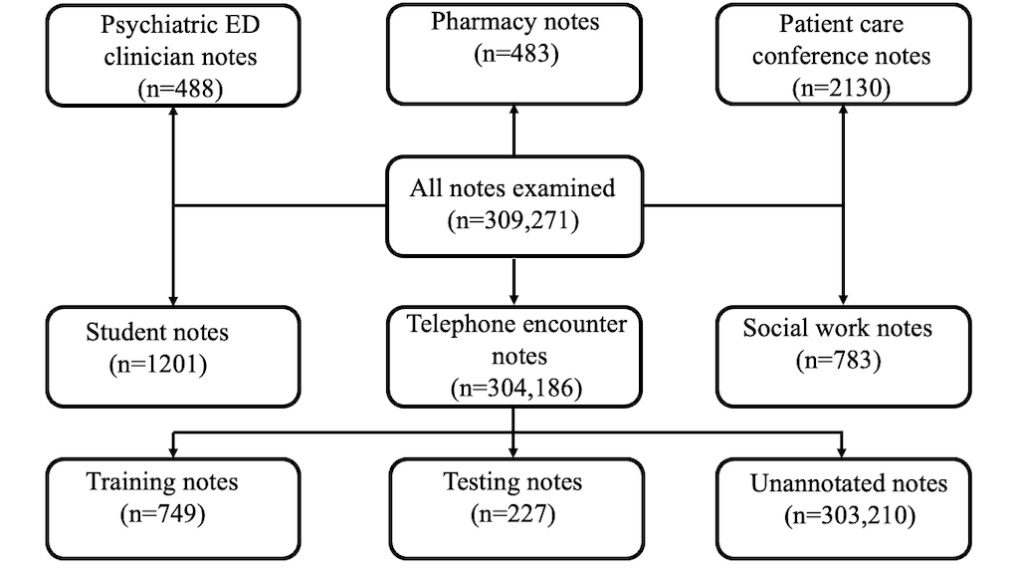
Schematic flow diagram (source: 2016-2019 Michigan Medicine Electronic Medical Records). ED: emergency department.

There are 60 different types of medical notes in MM. We randomly explored 10 notes from each category to identify the most promising type of notes for this study. Moreover, we sought expert advice from a geriatric MM nurse to identify the most promising medical notes for information about caregivers. Both approaches led us to use *telephone encounter* notes. Out of 2205 unique patients, 2017 had at least one telephone encounter note. We randomly selected and annotated a total of 976 telephone encounter notes (n=224 unique number of patients), of which 749 (77%) and 227 (23%) notes were partitioned into training (n=167 unique number of patients) and test (n=57 unique number of patients) sets, respectively. Furthermore, we ensured that all notes for each patient were kept within the same set. Figure 1 presents a schematic flow diagram of our sampling process.

### Annotations

To accomplish high interrater reliability, 2 team members, a nurse experienced in reading and writing medical notes at MM and a social scientist with no medical background, independently annotated all notes. Discrepancies in annotation were resolved with all team members’ participation. Our research questions included the following:

1. Does the patient reside at home or in an institution?
2. Does the patient have a formal (hired) caregiver?
3. Does the patient have an informal (a family member or a friend) caregiver?

The above research questions were chosen because the place of residence and caregiver availability are interconnected. Furthermore, our caregiver features were not mutually exclusive because it is plausible that the patient has both an informal and formal caregiver simultaneously [9]. Each criterion had the following two levels for annotation: 0 (based on lack of information or explicit negation) and 1 (based on implied or explicit narration in the note). If the note had no information about potential outcomes, we coded all features as zeros. Since a patient’s circumstances (place of residence and caregiver availability) may change over time, our unit of analysis was the note (nested within individual patients). Each note was annotated independently, relying only on information found in that note. Using this method, we sought to identify the patient’s place of residence and caregiver availability longitudinally at each specific time.

### Model

First, we preprocessed the data based on patterns we saw in the training set and then used 2 lexicons to construct a rule-based approach to characterizing each note. We measured the model’s performance in training and test sets, separately, using the *F*_1_-score, accuracy, sensitivity, and specificity of the model against our gold standard—manual annotations of the notes. *F*_1_-score summarizes the predictive power of an algorithm as the harmonic mean of precision and recall. Accuracy measures how many observations—positive and negative—were correctly classified [20].

### Preprocessing

Through our annotation process, we discovered multiple terms that frequently led to false positives. For example, “family medicine” raised a false positive for “family,” and “patient portal” raised a false positive for “patient.” Additionally, some notes contain template sections and subheading phrases such as “Family History,” which would list multiple familial relations that, in this context, would not be caregivers. These words and phrases were removed from medical notes before applying our algorithm (items 3 and 4 in the “Description of Rule-Based Algorithm” below).

### Lexicon

Our 2 lexicons were dictionaries of terms used to identify (1) place of residence and (2) type of caregiver, if any. Specific terms (eg, “home,” “atria ann arbor,” and “linden square”) were used to determine the current place of residence. To determine if a patient resides in an institution or at home, we used a list of nursing homes and care facilities in Washtenaw County (obtained from the University of Michigan) and a list of skilled nursing facilities in the state of Michigan (obtained from the Centers for Medicare and Medicaid Services website) [21]. Caregiver type was categorized into 2 groups—formal and informal—using general terms for caregivers (eg, “sister,” “husband,” or “visiting nurse”). To determine the presence and type of caregivers, we used a list of commonly used terms for friends and family members and formal caregivers based on our consultations with practitioners (nurses, physicians, and care coordinators at MM).

Our data dictionary can be found in alphabetical order in Table S2 in Multimedia Appendix 1. Should a term from the dictionary be found in a medical note, we would use the corresponding labels associated with the term to characterize the note. For example, if a “visiting nurse” and “spouse” were both found in a note, the algorithm would rule that the patient had both formal (visiting nurse) and informal (spouse) caregivers.

Multiple rules were implemented to account for a more complex logic in determining the place of residence and caregiver presence or type. Throughout the algorithm, a 4-word window, rather than a fewer- or more-word window, was used because the 4-word window achieved the best accuracy in the training set.

#### Patient Verb Neighborhood

A lexicon was created that included verbs such as “agreed,” “asked,” or “reported,” suggesting that a patient resides at home if these verbs appeared within a 4-word window of the following terms: “pt,” “patient,” or “patient’s” (Table S3 in Multimedia Appendix 1). This was used to determine whether the patient had any relevant caregiver information, even if identified terms in the dictionary were not found in the note. Since health care workers would often discuss the “patient” in a non–caregiver related context, we could not simply search for the previous 3 words.

#### Institution Negation Neighborhood

In many cases, an institution’s name appears in the note without any relation to patient’s current place of residence or status of caregiver support. Examples include potential plans when a patient was discharged from the hospital or in discussion with family members for referral to an institution or if a patient was in an institution for subacute rehabilitation, which would not be considered a place of residence or a caregiver. We created a lexicon for institution negation words and searched for them within a 4-word window of each institution’s name. If the specified negation words were found, then the institution was disregarded for that note.

If no terms were found, it was determined that the note had no information available to predict the place of residence or caregiver presence, and all fields were set to zero.

### Description of the Rule-Based Algorithm

The 13 selection criteria, provided in the annotation guidelines and coded in order in the algorithm, are described below:

1. Replace “PT” (uppercase) with “physical therapy” in the original medical notes to avoid erroneous pickups of “pt” as an abbreviation of “patient.”
2. Convert the original text in notes to lowercase letters.
3. Remove the following patterns in the lowercase notes to avoid false positives: “nurse navigator,” “navigator nurse,” “patient portal,” “patient name,” “relationship to patient” followed by blank spaces with no answer, “e.g., visiting nurse,” “patient & caregiver,” “patient or caregiver,” “patient &/or caregiver,” “family medicine,” “family practice,” “family doctor,” “family physician,” “alone with family,” “verbalizes understanding,” and “verbalized understanding.”
4. To avoid falsely labeling “family” as informal caregivers, we removed each occurrence of “family history” and all words that follow until a new line character.
5. Remove all occurrences of “aid” and “aids” in a lowercase note when any of the following shows up: “sleeping aid,” “sleeping aids,” “sleep aid,” “sleep aids,” “hearing aid,” “hearing aids,” “hear aid,” and “hear aids.”
6. Substitute the following patterns with “patient” to avoid falsely picking up “want” in the proximity of “patient” or “pt”: “want the patient,” “wants the patient,” “wanting the patient,” “want the pt,” “wants the pt,” “wanting the pt,” “want pt,” “wants pt,” and “wanting pt.”
7. Substitute “pt or ot,” “pt and ot,” or “pt/ot” with “physical therapy and ot” to avoid falsely picking up “pt” as “patient.”
8. Substitute “e-mail” with “email” before tokenization to avoid “e-mail” being split into “e” and “mail.”
9. Substitute variants of “patient partner” (eg, “patient’s partner,” “patients partner,” and “patient\ns partner” with “\n” being a new line character) with itself.
10. Oftentimes, the evidence of a “visiting nurse” may present itself as a variant (eg, “visit from a home care nurse”). To avoid missing such cases, for each sentence containing “nurse,” we searched for variants of “visiting” (eg, “visit”) before the occurrence of “nurse” within the sentence or searched for variants of “visiting” after the occurrence of “nurse” within that sentence and the sentence that follows.
11. To avoid falsely labeling informal caregiver or home when an institutional caregiver is present, in each lowercase medical note, we removed all occurrences of “patient,” “pt,” “care giver,” “caregiver,” and “guardian” in any sentence that included an institutional n-gram.
12. If any institutional term in the dictionary showed up in the note, there is evidence of institutional caregiver. To rule out false positives as potential, past, or unapproved institutional caregivers or when the service is for rehabilitation purpose only (eg, “patient discharged from Glacier Hills,” “returned home from Glacier Hills,” and “on the waiting list for Glacier Hills”), we looked for variants of “return from” (eg, “returning from” and “returned from”), “discharged from” (eg, “discharges from” and “discharging from”), “waiting list” (eg, “wait list” and “waitlist”), “cancel,” “decline,” “approve,” require,” “suggest,” “request,” and “rehabilitation” (eg, “rehab”) in the sentence containing an institutional term. If any of the variants shows up in the sentence, all occurrences of the institutional term would be disregarded in the note.
13. To see whether there is evidence of home self-care (home=1) when an institutional caregiver is absent, we looked for patient verbs within a prespecified (n=4) word window of “patient,” “pt,” or “patient’s.” If there is at least one patient verb within a certain neighborhood, there is evidence that the patient is involved in the decision-making of their own health to some extent. In addition, the occurrence of “relationship to patient:” followed by “patient,” “pt,” or “self” in the note also constitutes evidence of home self-care.

### Potential Reasons for Misclassification

The main reasons for algorithm misclassifications using annotated medical notes as our gold standard will be discussed and summarized.

### Generalizability Using Other Medical Notes

To test the generalizability of our algorithm in other notes, we examined what percentage of the data dictionary features can be found in other medical notes. None of these notes were annotated. The findings provided some preliminary data for the next step, which is using other medical notes to make the algorithm more generalizable.

### Ethics Approval

This research is approved by Michigan Medicine’s Institutional Review Board (HUM00129193).

## Results

We used R package version 3.6.3 (The R Foundation) to develop and test our NLP algorithm. Figure 1 presents the schematic flow diagram of our sampling. Out of the 304,186 available telephone encounter notes for our patient cohort, we annotated 749 notes for training and 227 notes for testing.

Table 1 displays some of the characteristics of our patient cohort (n=223). The average age was 78 (SD 10.94) years, with the majority being female (n=128, 57%). About 79% (n=176) were of White and 15% (n=33) of Black racial backgrounds. The average length of stay in the hospital was 6.78 (SD 6.54) days. Approximately 24% (n=54) of our patient population were readmitted or died within 30 days after discharge from the hospital.

Table 2 shows the results of our rule-based algorithm in our training and test sets. Among our features of interest (residing at home, residing at an institution, having a formal caregiver, and having an informal caregiver), identifying an informal caregiver was the most reliable feature. The result from our test set indicates high levels of accuracy and reliability for identifying an informal caregiver (*F*_1_=0.942, accuracy=0.947, sensitivity=0.970, and specificity=0.928). Identifying whether the patient lives at an institution was the least reliable measure, with the algorithm being prone to false positives (*F*_1_=0.638, accuracy=0.899, sensitivity=0.512, and specificity=0.978). The overall accuracy level for all 4 features in training and test sets were 0.858 and 0.655, respectively.

Table 3 summarizes the potential causes of misclassification, along with some examples and plausible explanations. The most common errors were related to (1) incomplete or misspelled names of the facilities; (2) past, uncertain, or undecided situations; (3) lack of specificity; (4) use of uncommon abbreviations; and (5) irregular use of templates.

To examine the generalizability of our algorithm using other medical notes, we measured the percentage of the features defined in our data dictionary in 5 different types of medical notes (patient care conference, pharmacy, psychiatric ED clinician, social work, and student) for our patient cohort (Table 4). The results indicate the highest level of generalizability for the informal caregiver. For example, 83% (n=1768) and 76% (n=595) of “patient care conference” and “social work” notes included information about informal caregivers. On the other hand, about 69% (n=333) of “pharmacy” notes had extractable information about a formal caregiver. This information can be used in future work to examine other types of medical notes.

**Table 1:** Descriptive characteristics of individuals included in training and test sets (N=223^a^).

Characteristics		Values
Age at the time of hospital admission, mean (SD)		77.96 (10.94)
**Sex, n (%)**		
	Female	128 (57.4)
	Male	95 (42.6)
**Race or ethnicity, n (%)**		
	White	176 (78.9)
	Black	33 (14.8)
	Hispanic	3 (1.4)
	Others	11 (4.9)
Length of stay in hospital, mean (SD)		6.78 (6.54)
**Payor, n (%)**		
	Medicare+private	103 (46.2)
	Medicare+Medicaid	34 (15.3)
	Medicare only	53 (23.8)
	Private only	6 (2.7)
	Others or missing	27 (12.1)
Readmitted or died within 30 days after hospital discharge, n (%)		54 (24.2)

^a^Number of unique individuals in the sample. Each person has one or more “Telephone Encounter” medical notes.

**Table 2:** Model performance summary for training and test sets.

Model	Training (N=749)						Test (N=227)				
	Place of residence			Caregiver			Place of residence			Caregiver	
	Home	Institution	Formal		Informal	Home		Institution	Formal		Informal
*F*_1_^a^	0.942	0.675	0.746		0.951	0.873		0.638	0.640		0.942
Accuracy^b^	0.923	0.964	0.923		0.964	0.837		0.899	0.841		0.947
Sensitivity^c^	0.947	0.609	0.680		0.971	0.870		0.512	0.571		0.970
Specificity^d^	0.875	0.987	0.971		0.960	0.778		0.978	0.930		0.928

^a^*F*_1_-score: the predictive power of an algorithm as the harmonic mean of precision and recall. *F*_1_-score ranges between 0 and 1, and the closer it is to 1, the better. *F*_1_-score=2 * (precision*recall) / (precision + recall).

^b^Number of observations, both positive and negative, correctly classified. Accuracy = (true positive + true negative) / (true positive + false positive + true negative + false negative).

^c^Ability of the model to predict a true positive of each category.

^d^Ability of the model to predict a true negative of each category.

**Table 3:** Potential causes of misclassification with explanation and examples.

Cause of error	Example	Explanation
Incomplete or misspelled names	“Pt stated that the nurse from Residential had difficulty drawing her blood.” “Medications are managed by staff at Gilbert House.” “Hartland of Ann Arbor”	Residential is short for “Residential Home Health.” If we add only “Residential” to our data dictionary, the false positive would increase. Gilbert House is not in the dictionary. Formal name is Gilbert Residence. Hartland is not in the dictionary. Formal name is Heartland Health Care Center.
Past, uncertain, or undecided situations	Will also need “in Home Care” order. “He shares that he has explored home health agencies (found them to be not suitable to what he is seeking).” “I love that her long-term goal is already established, and Glacier Hills is her final choice.” “Will have a visit nurse in the near future (will be at sister’s house).”	“Home care” picked up by NLP as formal=1. Falsely picked up “home health” as formal=1. Falsely picked up institution=1 and formal=1. Falsely picked up visiting nurse as formal=1.
Lack of specificity	“Spoke with Donna who was caring for Mr. xxx.” “Ellen manages medications using monthly organizer.” “Calling from rehab facility and has some questions regarding wound care.”	It is not clear whether “Donna” is a formal or informal caregiver. The algorithm picked up formal=1. Algorithm missed Ellen as a formal caregiver (formal=0). In some cases, patient stays in the rehab facilities (institution=1 and formal=1), and in some cases, patient stays at home and goes to the rehab facility (institution=0 and formal=0). Due to this ambiguity, we did not include rehab facility in the dictionary.
Uncommon abbreviations	“pt’s dtr” “her dau is working during the day.”	dtr and dau are short for daughter. They were not listed in the dictionary.

**Table 4:** Results of the natural language processing caregiver algorithm in other medical notes (the results show what percentage of the data dictionary features can be found in other medical notes).

Note type	Count, n	Overall, n (%)^a^	Resides at home, n (%)	Resides in an institution, n (%)	Formal caregiver, n (%)	Informal caregiver, n (%)
Telephone encounter	2000	1744 (87.2)	1326 (66.3)	426 (21.3)	704 (35.2)	1612 (80.6)
Patient care conference	2130	1825 (85.7)	1442 (67.7)	481 (22.6)	688 (32.3)	1768 (83.0)
Pharmacy	483	411 (85.0)	128 (26.4)	320 (66.2)	333 (68.9)	140 (29.0)
Psychiatric ED^b^ clinician	488	351 (71.9)	394 (80.7)	41 (8.4)	55 (11.3)	345 (70.7)
Social work	783	621 (79.3)	612 (78.2)	147 (18.8)	212 (27.1)	593 (75.7)
Student	1201	921 (76.7)	873 (72.7)	160 (13.3)	240 (20.0)	852 (70.9)

^a^The overall percentage represents the proportion that at least one of the features in our data dictionary was used in the listed medical notes, while the feature-specific percentage indicates the proportion of notes containing information regarding the specific outcome. This was done to test the generalizability of the algorithm in other medical notes for future work.

^b^ED: emergency department.

## Discussion

### Principal Findings

This project was the first to assess whether medical notes can be used to identify caregiver availability and place of residence. We used a rule-based NLP algorithm on a subset of telephone encounter notes recorded between 2016 and 2019 for patients diagnosed with dementia to determine caregiver availability (formal and informal) and place of residence (home or institution). Our algorithm reliably identified the availability of an informal caregiver (*F*_1_-score=0.94), moderately identified home as a place of residence (*F*_1_-score=0.87), and poorly identified if the patient lives in an institution (*F*_1_-score=0.64) or has a formal caregiver (*F*_1_-score=0.64).

### Comparison With Prior Work

Hospitals and health systems have made, and continue to make, substantial investments in their EMR systems. Although a systematic collection of salient medical and social data remains a work in progress, successful efforts using NLP algorithm have enabled efficient mining of rich free-text medical notes for various risk assessment or decision-making tools aimed at reducing the occurrences of adverse health events and wasteful spending [22-24]. Our study aligns with this work to identify caregiver availability for patients whose well-being depends on caregivers.

For many older patients, especially people with cognitive decline or disability, information on caregiver availability has numerous applications. For instance, a recent work by Choi et al [9] reveals that among people with dementia or disability, those with a greater number of informal caregivers (ie, family members or close friends) are less likely to be institutionalized. Availability of caregiver information in medical settings may inform a better care transition (ie, discharge planning from the hospital), care use (ie, institution vs home), and care costs [25-29]. Through care coordination with caregivers, patients may experience better adherence to follow-up appointments and more effectively follow a prescribed diet and medication regimen. Moreover, patients who need a caregiver but have little or no family support can be identified by social workers or care coordinators to proactively navigate the use of formal care (ie, nursing home or paid home care) [29-32].

For this study, we used telephone encounter notes, initiated based on phone conversations with patients or their caregivers. Most of these notes are written by nurses based on their conversations with patients or family members of the patient at different time points. Perhaps because telephone encounter notes were based on direct conversations with patients or family members (or other informal caregivers), the algorithm was highly accurate in identifying informal caregivers. Furthermore, since usually informal caregivers are close family members, we had a better data dictionary to identify them in text. On the contrary, considering the vast number of places that offer a range of services from adult day care centers to independent living, perhaps we overfitted the model in training set. Hence, there was a drop in accuracy for the other 3 variables in the test set. More work is needed to detect short- and long-term residential places or paid caregiver organizations or agencies.

Furthermore, in many cases, it was hard to manually—through human interpretation—decipher the notes. Medical notes either have no standard template or the existing templates were not standardized or used irregularly. Various health care professionals (residents, physicians, nurses, social workers, etc) with limited resources and under time pressure write these notes. Hence, nonstandardized abbreviations (ie, “dau” for “daughter”), spelling errors, and incorrect and uncommon names are used regularly. Many of these issues cannot be addressed using off-the-shelf packages or programs. By contrast, although not generalizable, the rule-based NLP algorithm served as a proof of concept for addressing many institution-specific terminologies. we plan to address many of the following limitations in our future work.

### Limitations, Strengths, and Future Work

Our study had a few noteworthy limitations. First, medical notes are based on unstructured text. We found large variations in the amount and type of information provided [31,32]. We used telephone encounter notes because, based on our examination of more than 60 different medical notes created within our institution, they provided the most relevant information regarding caregivers. We had, however, reasonable results detecting at least some elements of our data dictionary in other notes. In the future, we plan to make our algorithm generalizable by training and validating it using other medical notes and data from other health care centers. Second, manual annotation of the notes is resource intensive. Thus, our sample size was relatively small, which we plan to expand in the future. We will also explore the use of more sophisticated and unsupervised machine learning algorithms. Third, to make the algorithm more straightforward, we did not distinguish between a lack of objective and negative evidence. Thus, if there was no evidence about a caregiver or place of residence, we marked that outcome as zero. In our future work, we plan to make the algorithm more granular by identifying how many of the notes had (1) a positive indicator, (2) a negative indicator, and (3) no indicator. Further, to identify whether the patient lived in an institution, we used a list of skilled nursing facilities provided by the Centers for Medicare and Medicaid Services. There are, however, many unlisted independent living centers, adult day care centers, and other facilities designed to provide various services (residential and otherwise). It is challenging to include a comprehensive list of these facilities and their services. Having a reliable national directory of these facilities would help improve the model’s accuracy in determining whether a patient lives in a facility or is the recipient of paid or formal services. Finally, in this exploratory work, we only examined the binary availability of caregivers. Our future work will be focused on more critical information such as the caregiver’s proximity to the patient, the days and times of availability, the caregiver’s relation with the patient, and their capacity to help.

### Conclusion

In this study, we used a rule-based approach to train, test, and develop an NLP algorithm using telephone encounter notes from our institution to identify whether a patient has a formal and informal caregiver and whether the patient resides at home or in an institution at each point in time. Our validated test results show high levels of accuracy and reliability, particularly in identifying whether a patient has an informal caregiver. This information is critical for vulnerable patient populations such as those with dementia. Our algorithm can be used as a stand-alone module or in conjunction with other tools to identify caregiver availability among high-risk patient populations. Future work will focus on making the algorithm more granular and generalizable so it can be used at other institutions.

## Appendix

Multimedia Appendix 1 Supplemental Tables 1-3.

## Abbreviations

EMR: electronic medical record
MM: Michigan Medicine
NLP: natural language processing

## Data Availability

Since medical notes contain identifiable patient information, data sharing is not applicable. The data dictionary is available in the web-based appendix. The natural language processing algorithm is also available [33].
