# Supplementary material for: Identifying Caregiver Availability Using Medical Notes With Rule-Based Natural Language Processing: Retrospective Cohort Study
Source: JMIR Aging. 2022 Sep 22;5(3):e40241. doi: 10.2196/40241 (PMC9539648; doi:10.2196/40241)
Supplement: Multimedia Appendix 1 [file aging_v5i3e40241_app1.docx]

Table S1. Diagnostic Codes for Alzheimer’s Disease and Related Dementia

|  |  | ICD10 Codes |
| --- | --- | --- |
|  | |  |
|  | **Alzheimer’s Disease and Related Dementia** | F0150, F0151, F0280, F0281, F0390, F0391, G300, G301, G308, G309 |

Table S2. Data Dictionary

|  | Lives at Home | Lives at an Institution | Have a Formal Caregiver | Have an Informal Caregiver |
| --- | --- | --- | --- | --- |
| a&r premiere care | 0 | 1 | 1 | 0 |
| aid | 1 | 0 | 1 | 0 |
| aide | 1 | 0 | 1 | 0 |
| al | 0 | 1 | 1 | 0 |
| alone | 1 | 0 | 0 | 0 |
| american house | 0 | 1 | 1 | 0 |
| american house senior living | 0 | 1 | 1 | 0 |
| arbor care on independence | 0 | 1 | 1 | 0 |
| assisted facility | 0 | 1 | 1 | 0 |
| assisted living | 0 | 1 | 1 | 0 |
| assisted living facility | 0 | 1 | 1 | 0 |
| atria | 0 | 1 | 1 | 0 |
| atria ann arbor | 0 | 1 | 1 | 0 |
| balfour | 0 | 1 | 1 | 0 |
| balfour senior living | 0 | 1 | 1 | 0 |
| barton manor | 0 | 1 | 1 | 0 |
| bateson | 0 | 1 | 1 | 0 |
| birchwood | 0 | 1 | 1 | 0 |
| birchwood estate | 0 | 1 | 1 | 0 |
| birchwood manor | 0 | 1 | 1 | 0 |
| birchwood retreat | 0 | 1 | 1 | 0 |
| board and care home | 0 | 1 | 1 | 0 |
| botsford commons | 0 | 1 | 1 | 0 |
| botsford commons senior community | 0 | 1 | 1 | 0 |
| botsford commons senior living community | 0 | 1 | 1 | 0 |
| brecon | 0 | 1 | 1 | 0 |
| brecon village | 0 | 1 | 1 | 0 |
| brecon village retirement community | 0 | 1 | 1 | 0 |
| brookdale | 0 | 1 | 1 | 0 |
| brookhaven | 0 | 1 | 1 | 0 |
| brookhaven manor retirement community | 0 | 1 | 1 | 0 |
| brooksdale | 0 | 1 | 1 | 0 |
| brother | 1 | 0 | 0 | 1 |
| campbell home | 0 | 1 | 1 | 0 |
| care giver | 1 | 0 | 0 | 1 |
| caregiver | 1 | 0 | 0 | 1 |
| cedar woods | 0 | 1 | 1 | 0 |
| cedarwoods | 0 | 1 | 1 | 0 |
| cedarwoods assisted living | 0 | 1 | 1 | 0 |
| chelsea retirement community | 0 | 1 | 1 | 0 |
| chelsea retirement community kresge health care center | 0 | 1 | 1 | 0 |
| chelsea retirement community towsley village memory loss care center | 0 | 1 | 1 | 0 |
| cherywood | 0 | 1 | 1 | 0 |
| clara's house | 0 | 1 | 1 | 0 |
| clare bridge | 0 | 1 | 1 | 0 |
| clare bridge ann arbor a brookdale community | 0 | 1 | 1 | 0 |
| continuing care returement community | 0 | 1 | 1 | 0 |
| crc | 0 | 1 | 1 | 0 |
| daughter | 1 | 0 | 0 | 1 |
| daughter in law | 1 | 0 | 0 | 1 |
| day care | 1 | 1 | 1 | 0 |
| daycare | 1 | 1 | 1 | 0 |
| discharge to home | 1 | 0 | 0 | 0 |
| discharged from hospital | 1 | 0 | 0 | 0 |
| discharged home | 1 | 0 | 0 | 0 |
| discharged to home | 1 | 0 | 0 | 0 |
| ecf | 0 | 1 | 1 | 0 |
| eisenhower center | 0 | 1 | 1 | 0 |
| eisenhower ctr | 0 | 1 | 1 | 0 |
| emmanuel house | 0 | 1 | 1 | 0 |
| evangelical | 0 | 1 | 1 | 0 |
| evangelical home | 0 | 1 | 1 | 0 |
| evangelical home saline/the redies center for rehabilitation & healthy living | 0 | 1 | 1 | 0 |
| evh | 0 | 1 | 1 | 0 |
| extended care facility | 0 | 1 | 1 | 0 |
| family | 1 | 0 | 0 | 1 |
| family friend | 1 | 0 | 0 | 1 |
| family member | 1 | 0 | 0 | 1 |
| father | 1 | 0 | 0 | 1 |
| friend | 1 | 0 | 0 | 1 |
| from the patient | 1 | 0 | 0 | 0 |
| gh | 0 | 1 | 1 | 0 |
| gilbert residence | 0 | 1 | 1 | 0 |
| gilbert residence | 0 | 1 | 1 | 0 |
| glacier hills | 0 | 1 | 1 | 0 |
| glacier hills care and rehabilitation center | 0 | 1 | 1 | 0 |
| glacier hills life care retirement community | 0 | 1 | 1 | 0 |
| glacier hills senior living community eva's house | 0 | 1 | 1 | 0 |
| glacier manor | 0 | 1 | 1 | 0 |
| glastonbury | 0 | 1 | 1 | 0 |
| golden acre residential care | 0 | 1 | 1 | 0 |
| grace | 0 | 1 | 1 | 0 |
| granddaughter | 1 | 0 | 0 | 1 |
| grandson | 1 | 0 | 0 | 1 |
| great lakes caring | 1 | 0 | 1 | 0 |
| group home | 0 | 1 | 1 | 0 |
| guardian | 1 | 0 | 0 | 1 |
| happiness sweet home | 0 | 1 | 1 | 0 |
| heartland | 0 | 1 | 1 | 0 |
| heartland health care center of ann arbor | 0 | 1 | 1 | 0 |
| hh | 0 | 1 | 1 | 0 |
| hillside | 0 | 1 | 1 | 0 |
| hillside terrace | 0 | 1 | 1 | 0 |
| hillside terrace retirement center | 0 | 1 | 1 | 0 |
| hillside terrace retirement community specialized/memory care | 0 | 1 | 1 | 0 |
| home aid | 1 | 0 | 1 | 0 |
| home care | 1 | 0 | 1 | 0 |
| home health | 1 | 0 | 1 | 0 |
| home nursing | 1 | 0 | 1 | 0 |
| homecare | 1 | 0 | 1 | 0 |
| homewatch | 1 | 0 | 1 | 0 |
| hospice | 1 | 0 | 1 | 0 |
| husband | 1 | 0 | 0 | 1 |
| in home help | 1 | 0 | 1 | 0 |
| in home services | 1 | 0 | 1 | 0 |
| independent living | 0 | 1 | 1 | 0 |
| inpatient rehabilitation facility | 0 | 1 | 1 | 0 |
| institution | 0 | 1 | 1 | 0 |
| irf | 0 | 1 | 1 | 0 |
| kennedy care | 1 | 0 | 1 | 0 |
| kisselburg | 0 | 1 | 1 | 0 |
| kisselburg home | 0 | 1 | 1 | 0 |
| life care community | 0 | 1 | 1 | 0 |
| linden | 0 | 1 | 1 | 0 |
| linden square | 0 | 1 | 1 | 0 |
| linden square assisted living center | 0 | 1 | 1 | 0 |
| living facility | 0 | 1 | 1 | 0 |
| long term care facility | 0 | 1 | 1 | 0 |
| ltcf | 0 | 1 | 1 | 0 |
| lurie terrace | 0 | 1 | 1 | 0 |
| managed by patient | 1 | 0 | 0 | 0 |
| managed by the patient | 1 | 0 | 0 | 0 |
| marquette house | 0 | 1 | 1 | 0 |
| meadow view assisted living | 0 | 1 | 1 | 0 |
| medical foster home program | 0 | 1 | 1 | 0 |
| memory support center at brecon village | 0 | 1 | 1 | 0 |
| mill pond manor | 0 | 1 | 1 | 0 |
| mother | 1 | 0 | 0 | 1 |
| mvn | 1 | 0 | 1 | 0 |
| native american medicine man | 1 | 0 | 0 | 1 |
| neighbor | 1 | 0 | 0 | 1 |
| nephew | 1 | 0 | 0 | 1 |
| niece | 1 | 0 | 0 | 1 |
| nursing facility | 0 | 1 | 1 | 0 |
| nursing home | 0 | 1 | 1 | 0 |
| occupational therapy | 0 | 1 | 1 | 0 |
| orchard hills manor | 0 | 1 | 1 | 0 |
| paid caregiver | 1 | 0 | 1 | 0 |
| patient partner | 1 | 0 | 0 | 1 |
| patient unable to be reached | 1 | 0 | 0 | 0 |
| presidential care givers | 0 | 1 | 1 | 0 |
| regency | 0 | 1 | 1 | 0 |
| regency at bluffs park | 0 | 1 | 1 | 0 |
| regency at canton | 0 | 1 | 1 | 0 |
| regency at whitmore lake | 0 | 1 | 1 | 0 |
| relationship to patient patient | 1 | 0 | 0 | 0 |
| reported by the patient | 1 | 0 | 0 | 0 |
| residential care facility | 1 | 0 | 1 | 0 |
| residential facility | 1 | 0 | 1 | 0 |
| saline evangelical | 0 | 1 | 1 | 0 |
| saline evh | 0 | 1 | 1 | 0 |
| sanctuary | 0 | 1 | 1 | 0 |
| sanctuary at st. joseph's village | 0 | 1 | 1 | 0 |
| sasse | 0 | 1 | 1 | 0 |
| significant other | 1 | 0 | 0 | 1 |
| silver maples | 0 | 1 | 1 | 0 |
| sister | 1 | 0 | 0 | 1 |
| skilled nursing facility | 0 | 1 | 1 | 0 |
| snf | 0 | 1 | 1 | 0 |
| son | 1 | 0 | 0 | 1 |
| son in law | 1 | 0 | 0 | 1 |
| son's girlfriend | 1 | 0 | 0 | 1 |
| spouse | 1 | 0 | 0 | 1 |
| st. joe's village | 0 | 1 | 1 | 0 |
| story point | 0 | 1 | 1 | 0 |
| storypoint | 0 | 1 | 1 | 0 |
| superior woods | 0 | 1 | 1 | 0 |
| superior woods health care center | 0 | 1 | 1 | 0 |
| ul | 0 | 1 | 1 | 0 |
| unable to reach patient | 1 | 0 | 0 | 0 |
| university commons | 0 | 1 | 1 | 0 |
| university living | 0 | 1 | 1 | 0 |
| vibrant life | 0 | 1 | 1 | 0 |
| vibrant life senior living | 0 | 1 | 1 | 0 |
| villa | 0 | 1 | 1 | 0 |
| villa at parkridge | 0 | 1 | 1 | 0 |
| visiting np | 1 | 0 | 1 | 0 |
| visiting nurse | 1 | 0 | 1 | 0 |
| visiting nurses | 1 | 0 | 1 | 0 |
| visiting rn | 1 | 0 | 1 | 0 |
| vn | 1 | 0 | 1 | 0 |
| widow | 1 | 0 | 0 | 0 |
| widowed | 1 | 0 | 0 | 0 |
| wife | 1 | 0 | 0 | 1 |
| woodbury | 0 | 1 | 1 | 0 |
| woodbury gardens | 0 | 1 | 1 | 0 |

Table S3. Patient Verbs

| **patient_verbs** |
| --- |
| accept |
| agree |
| ask |
| call |
| chose |
| confirm |
| contact |
| decide |
| denie |
| deny |
| encourage |
| engage |
| inform |
| manage |
| obtain |
| re-iterat |
| reiterat |
| report |
| request |
| say |
| speak |
| spoke |
| stat |
| understand |
| verbaliz |
| want |
| email |
| e-mail |
| complain |
| notif |
| describe |
